# Incidence and mortality trends of primary cutaneous melanoma: A 50-year Rochester Epidemiologic Project study

**DOI:** 10.1016/j.jdin.2024.04.010

**Published:** 2024-05-10

**Authors:** Jacob P. Reinhart, Elliott H. Campbell, Sydney L. Proffer, Olivia M. Crum, Austin Todd, Lawrence E. Gibson, Jerry D. Brewer, Addison M. Demer

**Affiliations:** a Department of Dermatology, Mayo Clinic School of Graduate Medical Education, Rochester, Minnesota; b Division of Clinical Trials and Biostatistics, Department of Quantitative Health Sciences, Mayo Clinic, Rochester, Minnesota; c Division of Dermatopathology, Department of Dermatology, Mayo Clinic, Rochester, Minnesota; d Division of Dermatologic Surgery, Department of Dermatology, Mayo Clinic, Rochester, Minnesota

**Keywords:** age distribution, epidemiology, female, incidence, male, melanoma, morbidity, mortality, overdiagnosis, skin cancer, skin neoplasms, survival rate

## Abstract

**Background:**

National cancer reporting-based registry data, although robust, lacks granularity for incidence trends. Expert opinion remains conflicted regarding the possibility of melanoma overdiagnosis in the context of rising incidence without a corresponding rise in mortality.

**Objective:**

To characterize 10- and 50-year trends in melanoma incidence and mortality.

**Methods:**

Multicenter, population-based epidemiologic study utilizing the Rochester Epidemiology Project for Olmsted County, Minnesota residents diagnosed with melanoma from 01/01/1970 to 12/21/2020. Age- and sex-adjusted incidence and disease-specific mortality are calculated.

**Results:**

Two thousand three hundred ten primary cutaneous melanomas were identified. Current age- and sex-adjusted incidence rates increased 11.1-fold since 1970s (*P* < .001). Over the last decade, there is an overall 1.21-fold (*P* < .002) increase, with a 1.36-fold increase (*P* < .002) among females and no significant increase among males (1.09-fold increase, *P* < .329). Melanoma-specific mortality decreased from 26.7% in 1970s to 1.5% in 2010s, with a hazard ratio (HR) reduction of 0.73 (*P* < .001) per 5-year period. Increased mortality was associated with Breslow thickness (HR 1.35, *P* < .001), age at diagnosis (HR 1.13, *P* = .001) left anatomic site (HR 1.98, *P* = .016), and nodular histogenic subtype (HR 3.08, *P* < .001).

**Limitations:**

Retrospective nature and focused geographic investigation.

**Conclusion:**

Melanoma incidence has continued to increase over the past decade, most significantly in females aged 40+. Trend variations among age and sex cohorts suggests external factors beyond overdiagnosis may be responsible. Disease-specific mortality of melanoma continues to decrease over the last 50 years.

**Capsule Summary**

• Rising incidence rates of primary cutaneous melanoma are well-documented over the past 50 years, with conflicting expert opinion about the possibility of overdiagnosis.
• High-quality population-based epidemiologic data with long-term follow-up validates rising melanoma incidence and decreasing mortality. Trend divergence among sex and age cohorts indicates external influence beyond overdiagnosis.

## Introduction

The rising US incidence of melanoma over the past 50 years is well-documented.^1^, ^2^, ^3^, ^4^, ^5^, ^6^ According to the Surveillance, Epidemiology, and End Results (SEER) Program, cutaneous melanoma is the fifth most common cancer diagnosis (excluding nonmelanoma skin cancer) in the United States, with an estimated lifetime risk of 2.1%.^1^ In recent years, overall incidence of melanoma has continued to rise an estimated 1.2% per year from 2010 to 2019.^1^ Among young adults (<age 40-49), however, there is evidence of stabilizing or decreasing incidence.^6^, ^7^, ^8^

Melanoma poses an estimated 5-year relative mortality rate of 6.3%.^1^ Although mortality trends had previously remained largely stable, some reports tracking SEER data between 2009 and 2018 indicate decreasing mortality between 17.9% and 30%.^2^^,^^9^

Despite the crucial nature of national registries such as the SEER database, delays and underreporting are inherently prevalent.^10^ Furthermore, SEER registries cover only 35% of the US population.^11^ Accordingly, high-quality population-based studies are critical to strengthen the validity of national database findings.

Our institution previously published large epidemiologic studies on melanoma incidence and mortality trends across Olmsted County, Minnesota residents from 1970 through 2009.^12^, ^13^, ^14^ These data reinforced the continued rise in melanoma incidence across all age groups, but highest among women aged 40 to 60 years old (24-fold increase),^13^ and men aged 61+ (11-fold increase).^14^ Increasing survivorship trends from 1970 to 2010 were also reported.^12^, ^13^, ^14^

A 2021 article in the New England Journal of Medicine utilizing national database data argued that relative stability in disease-specific mortality with rising incidence signifies overdiagnosis.^15^ With this context, and in a landscape of significant therapeutic advancements for high-risk melanomas over the past decade, this study sought to provide a 10-year update to our team’s previously reported melanoma incidence and mortality data from 1970 to 2009.^12^, ^13^, ^14^

## Methods

### Patient selection

Patients aged 18+ with an initial lifetime diagnosis of primary cutaneous melanoma between January 1, 1970 and December 21, 2020 were identified using the Rochester Epidemiology Project (REP).

The REP is an aggregated multicenter medical record database for all residents of Olmsted County, Minnesota who obtain care from Mayo Clinic, Olmsted Medical Center, and other affiliated private practitioners. The comprehensive county-wide tracking features of the platform provide validity for epidemiologic studies.^16^ This patient population is largely non-Hispanic Caucasian with a comparable socioeconomic status to the entire United States and a population of 162,847 residents in 2020.^17^

REP patients were identified through ICD codes for malignant melanoma, followed by detailed manual chart review for verification. Patients with a prior history of cutaneous melanoma, those with ocular or mucosal melanoma, or those who denied research authorization were excluded.

Data collection identified melanoma tumor characteristics (Breslow thickness, histogenic subtype, anatomic site, and American Joint Committee on Cancer [AJCC] staging eighth edition), patient demographics, and mortality data. Individual death certificates were viewed to verify melanoma as a cause of death. Results were separated by sex, and 3 aged cohorts (18-39, 40-60, and 61+) which replicate our group’s previously reported data.^12^, ^13^, ^14^

### Statistical analysis

Data analysis was completed from March 2022 to October 2023. Age- and sex-specific incidence rates (per 100,000 person-years) in Olmsted County were calculated based off REP data and adjusted to the total population of the United States. The 95% CI for incidence rates were calculated assuming a Poisson error distribution. Differences in incidence rates by sex, age group, and calendar period were assessed by fitting Poisson regression models to the incidence counts using the natural logarithm of the total person-years as an offset.

Cause-specific (ie, death due to melanoma) mortality rates were estimated using the Kaplan-Meier method. Univariable and multivariable associations with the risk of death due to melanoma were evaluated using Cox proportional hazards regression models and summarized with hazard ratios (HRs) and 95% CIs. Variable selection for these models was determined based on clinical expertise, previous literature, and the univariable analysis. Statistical significance was defined at a *P* < .05. All statistical analyses were performed using RStudio Version 4.1.3 (RStudio).

## Results

### Patient demographics and tumor characteristics

A total of 2310 patients with an initial lifetime diagnosis of melanoma between January 1, 1970 and December 21, 2020 were identified. Tumor characteristics and patient demographics are reported in Table I. Across all patients, the trunk was the most affected anatomic site (803, 36.1%). Left-sided melanomas were more prevalent (1073, 46.5%) than those arising on the right side (999, 43.2%). Most melanomas were classified as invasive (1654, 71.6%) at the time of diagnosis, compared with in-situ disease (6949, 28.1%). The most common histogenic subtype was superficial spreading (1103, 66.7%), followed by lentigo maligna (198, 12.0%) and nodular (116, 7.0%). Histopathologic identification of a pre-existing nevus was reported in a minority of melanomas (237, 10.3%). Most melanomas were classified as AJCC V8 stage IA (1218, 73.5%).

**Table I:** Patient demographics and clinicopathologic characteristics of 2310 primary cutaneous melanomas in Olmsted County, Minnesota

Characteristic	Value
Age grouping	
18 to 39	422 (18.3%)
40 to 60	858 (37.1%)
61 +	1030 (44.6%)
Sex	
Female	1091 (47.2%)
Male	1219 (52.8%)
MM site	
Head/neck	439 (19.7%)
Lower limb	385 (17.3%)
Trunk	803 (36.1%)
Unknown/other	5 (0.2%)
Upper limb	594 (26.7%)
MM location	
Right	999 (43.2%)
Left	1073 (46.5%)
Central	195 (8.4%)
Not stated	43 (1.9%)
Preexisting nevus	
Absent	908 (39.3%)
Compound nevus	60 (2.6%)
Dermal nevus	67 (2.9%)
Nevus NOS	110 (4.8%)
Unknown	1165 (50.4%)
Clark level	
I	649 (28.1%)
II	790 (34.2%)
III	470 (20.3%)
IV	317 (13.7%)
V	29 (1.3%)
Unknown	55 (2.4%)
Invasive or in situ MM	
Invasive	1654 (71.6%)
In situ	649 (28.1%)
Insufficient info	7 (0.3%)
Among invasive MM only	
Histogenic type	
LM	198 (12.0%)
SS	1103 (66.7%)
Nodular	116 (7.0%)
Desmoplastic	12 (0.7%)
Spitzoid	5 (0.3%)
Acral lentiginous	19 (1.1%)
Mixed	9 (0.5%)
Subungual	1 (0.1%)
Other	16 (1.0%)
Unknown	175 (10.6%)
Breslow depth or thickness (in mm)	
Median	0.5
Pathologic stage (AJCC eighth edition)	
IA	1218 (73.5%)
IB	200 (12.1%)
IIA	56 (3.4%)
IIB	44 (2.7%)
IIC	10 (0.6%)
III	41 (2.5%)
IV	57 (3.4%)
Not documented	31 (1.9%)

*AJCC*, American Joint Committee on Cancer; *LM*, lentigo maligna; *MM*, malignant melanoma; *NOS*, not otherwise specified; *SS*, superficial spreading.

### Fifty-year incidence

**Fig 1:**
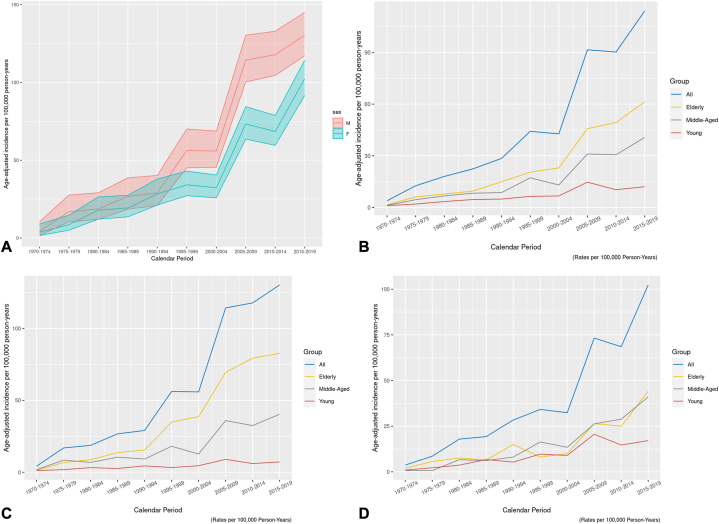
Age-adjusted incidence of primary cutaneous melanoma in Olmsted County, Minnesota. **A,** Overall, separated by sex. **B,** Overall, separated by aged cohorts (young = 18-39; middle-aged = 40-60; elderly = 61+). **C,** Males, separated by aged cohorts (young = 18-39; middle-aged = 40-60; elderly = 61+). **D,** Females, separated by aged cohorts (young = 18-39; middle-aged = 40-60; elderly = 61+).

Overall age- and sex-adjusted incidence rates increased by 11.1-fold (*P* < .001) from 8.44 (95% CI, 5.79-11.10) per 100,000 person-years in 1970-1979 to 93.75 (95% CI, 88.27 to 99.24) per 100,000 person-years in 2011 to 2020. Separated by sex, female incidence increased by 14-fold (*P* < .001) from 5.98 (95% CI, 3.31-8.66) to 83.77 (95% CI, 76.60-91.07) per 100,000 person-years. Incidence among males increased by 9.75-fold (*P* < .001) from 11.21 (95% CI, 6.37-16.05) to 109.32 (95% CI, 100.59-118.04) per 100,000 person-years (Fig 1, *A*).

### Ten-year incidence

Throughout the most recent 10-year period (2005-2009 vs 2015-2020), incidence has continued to increase significantly by 1.36-fold (*P* < .002) among females (71.73 vs 97.50 per 100,000 person-years). Male incidence demonstrated a statistically insignificant 1.09-fold increase (*P* < .329) over the same 10-year period (104.85 vs 114.21 per 100,000 person-years).

When separated by aged cohorts (Fig 1, *B*-*D*), adults aged 40 to 60 and 61+ continue to account for the highest overall incidence of melanoma. The greatest rise in incidence over the most recent 10-year period (2005-2009 vs 2025-2020) is observed in females aged 40 to 60 and 61+ (1.5-fold increase, *P* = .002; and 2.7-fold increase, *P* < .001 respectively). Additional data regarding aged cohorts is previously reported.^18^, ^19^, ^20^

### Mortality and survival

Melanoma-specific death was observed in 100 patients, accounting for an overall disease specific-mortality rate of 4.3%. Of those, 93 deaths occurred within 10 years following melanoma diagnosis. Disease-specific death was not observed for melanoma in-situ.

**Fig 2:**
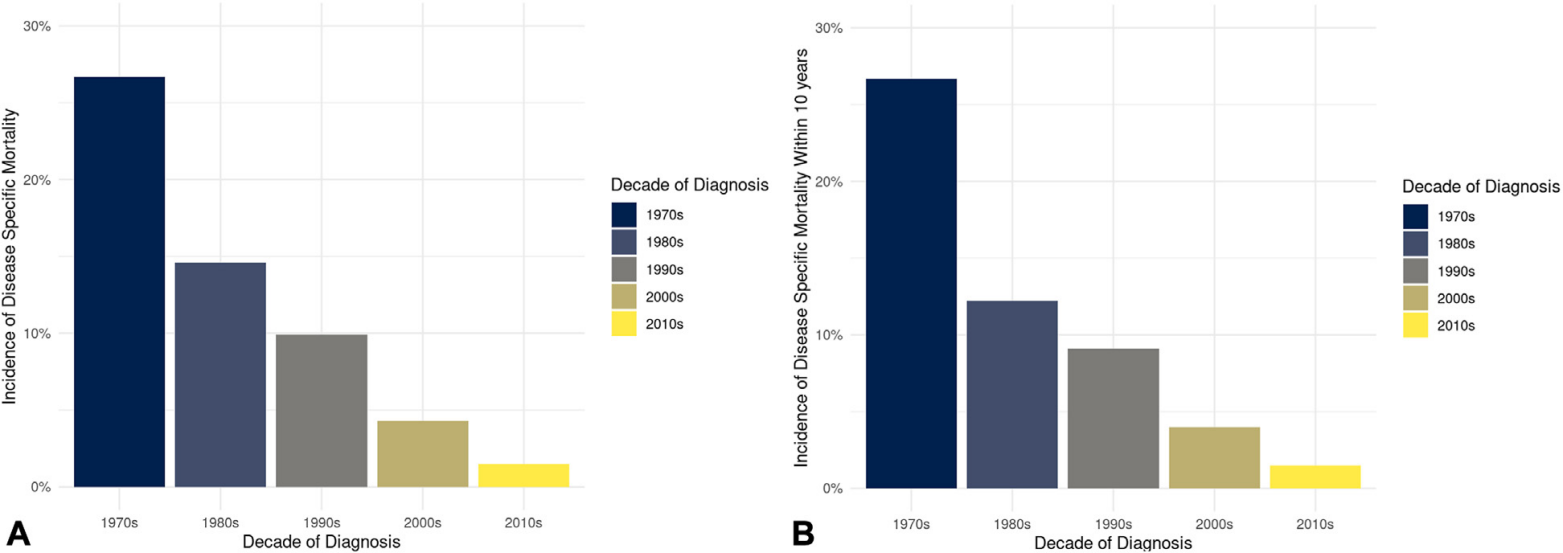
Disease-specific mortality of patients with primary cutaneous melanoma in Olmsted County, Minnesota. **A,** Overall mortality. **B,** Mortality within 10 years of diagnosis.

**Fig 3:**
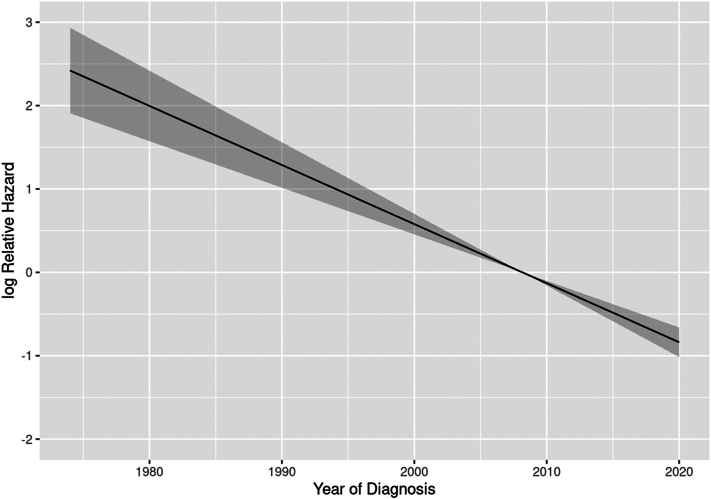
Hazard ratio for risk of death due to melanoma according to calendar year of diagnosis.

Fig 2, *A* depicts disease-specific mortality by decade of diagnosis. Death due to melanoma decreased with each progressive decade and a more recent diagnosis was associated with a lower disease-specific death due to melanoma (HR 0.73 per 5-year increase in calendar year of diagnosis (95% CI, 0.65-0.81, *P* < .001). To reduce bias regarding length of follow-up, disease-specific mortality within 10 years of diagnosis was calculated (Fig 2, *B*). A decrease in disease-specific death is further depicted in a HR plot according to year of diagnosis (Fig 3).

**Fig 4:**
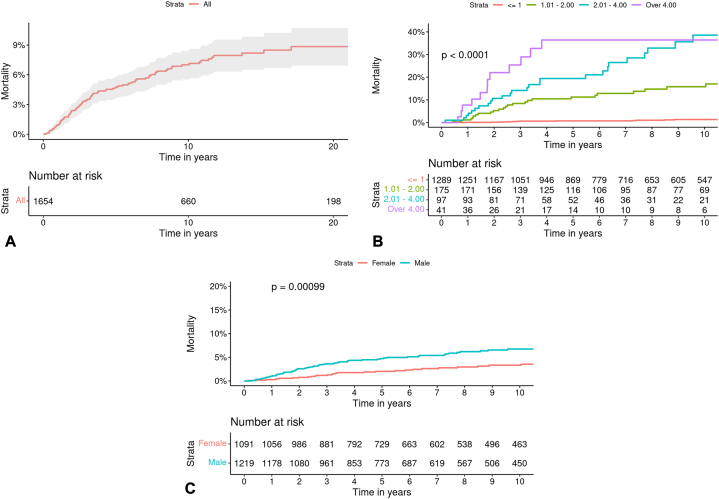
Projected disease-specific mortality following diagnosis of primary cutaneous melanoma. **A,** Overall mortality. **B,** Mortality according to Breslow thickness. **C,** Mortality according to sex.

Projected disease-specific mortality for invasive melanoma is represented by Kaplan-Meier curves in Fig 4. Survival estimates at 10- and 20-years following diagnosis was found to be 93% (95% CI, 91.6%-94.5%) and 91.2% (95% CI, 89.3%-93.1%), respectively.

Table II displays disease-specific mortality related to numerous variables, with univariable and multivariable HR calculations. According to multivariable analysis, an increase in disease-specific mortality was significantly associated with the following: higher age at diagnosis (HR 1.13 per 5-year increase in age, 95% CI, 1.05-1.22, *P* = .011), increased Breslow thickness (HR 1.34, 95% CI, 1.25-1.43, *P* < .001), left-sided anatomic site (HR 1.98, 95% CI, 1.14-3.34, *P* = .016), and nodular histogenic subtype (HR 3.08, 95% CI, 1.75-5.42, *P* < .001).

**Table II:** Univariable and multivariable models for disease-specific mortality of melanoma

Parent variable	Sub-variable	Value (percent)	Univariable HR (CI)	Multivariable HR (CI)
Year of diagnosis (5-year)	-	-	0.70 (0.65-0.76, *P* < .001)	0.73 (0.65-0.81, *P* < .001)
Age at diagnosis (5-year)	-	-	1.08 (1.02-1.15, *P* = .011)	1.13 (1.05-1.22, *P* = .001)
Sex	Female	1091 (47.2)	-	-
	Male	1219 (52.8)	2.00 (1.31-3.04, *P* = .001)	1.53 (0.91-2.56, *P* = .107)
Breslow thickness (mm)	Mean (SD)	0.9 (1.3)	1.35 (1.28-1.41, *P* < .001)	1.34 (1.25-1.43, *P* < .001)
Race	Non-White	51 (2.2)	-	-
	White	2259 (97.8)	1.39 (0.19-10.00, *P* = .741)	-
Laterality	Right	999 (43.2)	-	-
(Side of body)	Left	1073 (46.5)	2.12 (1.35-3.34, *P* = .001)	1.98 (1.14-3.45, *P* = .016)
	Central	195 (8.4)	1.13 (0.47-2.75, *P* = .781)	-
	Not Stated	43 (1.9)	6.49 (2.83-14.91, *P* < .001)	-
Pathologic stage (AJCC eighth edition)	I	1418 (87.7)	-	-
	II	100 (6.2)	12.37 (6.81-22.49, *P* < .001)	-
	III	41 (2.5)	20.49 (10.06-41.73, *P* < .001)	-
	IV	57 (3.5)	69.67 (42.03-115.48, *P* < .001)	-
Anatomic site (combined)	Head/Neck	439 (19.7)	1.68 (1.07-2.64, *P* = .025)	1.48 (0.83-2.66, *P* = .184)
	Other	1787 (80.3)	-	-
Anatomic site (detailed)	Trunk	803 (36.1)	-	-
	Head/Neck	439 (19.7)	1.85 (1.08-3.17, *P* = .025)	-
	Lower Limb	385 (17.3)	1.38 (0.77-2.49, *P* = .281)	-
	Upper Limb	594 (26.7)	0.85 (0.46-1.55, *P* = .589)	-
	Other/Unknown	5 (0.2)	57.85 (20.12-166.36, *P* < .001)	-
Histogenic subtype (combined)	Nodular	116 (7.0)	5.48 (3.50-8.57, *P* < .001)	3.08 (1.75-5.42, *P* < .001)
	Other	1538 (93.0)	-	-
Histogenic subtype (detailed)	LM	198 (12.0)	-	-
	SS	1103 (66.7)	5.91 (0.81-43.09, *P* = .080)	-
	Nodular	116 (7.0)	47.65 (6.47-351.17, *P* < .001)	-
	Desmoplastic	12 (0.7)	13.41 (0.84-214.51, *P* = .066)	-
	Spitzoid	5 (0.3)	0.00 (0.00-Inf, *P* = .996)	-
	Acral Lentiginous	19 (1.1)	20.06 (1.82-221.29, *P* = .014)	-
	Mixed	9 (0.5)	42.50 (3.85-468.78, *P* = .002)	-
	Other/Unknown	192 (11.6)	29.97 (4.09-219.60, *P* = .001)	-

*AJCC*, American Joint Committee on Cancer; *HR*, hazard ratio; *LM*, lentigo maligna; *MM*, malignant melanoma; *SD*, standard deviation.

**Fig 5:**
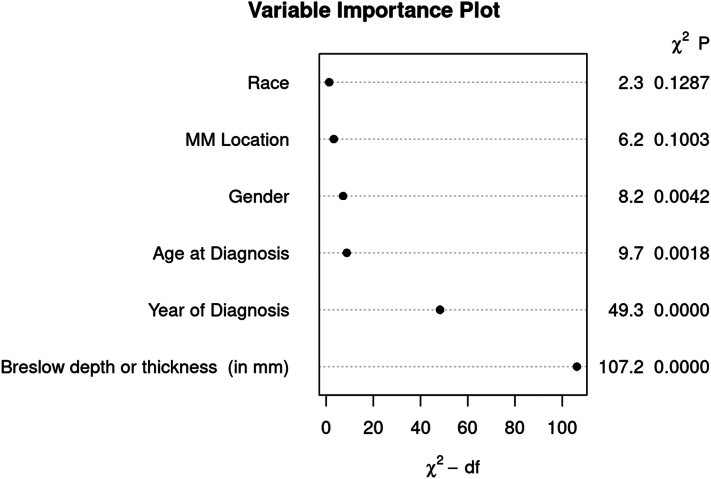
Variable importance plot depicting the relative importance of multiple variables according to disease-specific mortality of melanoma.

Characteristics associated with an increase in disease-specific mortality, but without statistical significance upon multivariable analysis include male sex (HR 1.53, 95% CI, 0.91 to 2.56, *P* = .107) and anatomic site of head or neck (HR 1.48, 95% CI, 0.83-2.66, *P* = .184). Increasing pathologic stage (AJCC eighth edition) was associated with statistically significant increase in disease-specific mortality upon univariable analysis but was not utilized for multivariable analysis. Caucasian race was not significantly associated with mortality (HR 1.39, *P* = .741). A variable importance plot depicting the relative importance of each variable according to disease-specific mortality is shown in Fig 5.

## Discussion

### Incidence

This 50-year epidemiologic study provides a 10-year update to previously reported melanoma incidence and mortality data from Olmsted County, Minnesota.^12^, ^13^, ^14^ Our results demonstrate a continued increase in age- and sex-adjusted melanoma incidence since 2009, which is largely consistent with recently reported incidence data showing continued increase in melanoma incidence among adults aged 40 years or older.^8^

Our findings demonstrate that males aged 61+ continue to have the highest overall incidence of melanoma, but that females aged 40+ are experiencing the greatest growth of incidence over the past decade. Female incidence remains higher than males among adults aged < 60. Furthermore, the slight rise observed in male incidence over the past 10 years was not statistically significant and suggests a potential stabilization of melanoma incidence among adult males.

It is important to address recent concerns for melanoma overdiagnosis, suggested from rising incidence rates without a corresponding rise in mortality.^15^^,^^21^^,^^22^ Multiple groups have attempted to analyze and quantify the magnitude of this proposed overdiagnosis.^23^^,^^24^ A 2024 ecological study examining the SEER database calculated an overestimation of melanoma diagnosis in 2018 of 49.7% in white men and 64.6% in white women, with an estimated 85.4% to 89.4% overdiagnosis for melanoma in situ.^24^ A 2022 cohort study used the trends in melanoma mortality among Black patients as a marker for improvements in medical care (a potential theory for declining mortality) and found an estimated overdiagnosis of melanoma in 59% to 60% of white patients.^23^ Despite using adept epidemiologic methods, the SEER database used in these studies presents inherent limitations with known delays, underreporting, and limited scope covering approximately 35% of the US population.^10^^,^^11^

Our data show converging rates among males and females overall, with stabilization in incidence of males of any age range and females <40 years of age. If overdiagnosis were the primary cause of rising incidence, a more uniform increase in melanoma rates across demographics might be expected. Instead, our data suggest that external demographic-specific risk factors and exposures may influence a rise in incidence among certain subsets of the population.

These findings are unable to confirm or deny the presence of overdiagnosis but support the multifactorial nature of melanoma incidence. Overdiagnosis alone would not fully account for the findings of our study which demonstrates the need to stratify population-based data into sex and age groups to fully elucidate incidence trends.

Importantly, the elevated use of tanning beds among females has been widely suggested as a prominent risk factor for melanoma.^25^, ^26^, ^27^ Exogenous female hormones have also been suggested as a melanoma risk factor, but with conflicting evidence.^28^ Ultimately, additional high-quality studies addressing risk factors for development of melanoma are warranted.

The present data demonstrating 10.3% of melanomas arising within a pre-existing nevus on histology is on the low end of previous reports ranging from 10.8%^29^ to 57.6%.^30^ Although this may suggest a higher rate of de novo melanomas, it should be noted that 50.4% of our pathology reports described pre-existing nevus as unknown. Importantly, these data cannot exclude loss of identifiable histopathologic features of a pre-existing nevus in late transformation of melanomas arising from pre-existing nevus.

The literature suggests a higher prevalence of left-sided melanomas,^31^^,^^32^ with 1 study of 6 population-based cancer registries showing a left:right (L/R) ratio of 1.10.^33^ Our study supports this prior data, with left-sided melanomas accounting for 46.5% of tumors, compared with 43.2% on the right (L/R ratio, 1.07). Increased left-sided ultraviolet exposure during vehicle driving has been suggested, but this does not explain the increased left-sided laterality of skin cancer in countries with vehicle driving on the right (Australia, England, Scotland) as seen in Brewster et al^33^ or for increased incidence of melanomas on the leg which may receive less UV exposure when driving.^34^ An additional theory includes the potential asymmetry of melanocytic embryonic development.^33^^,^^35^

### Mortality

Our findings show a decrease in melanoma-specific mortality over the past decade, and a continued decline over the past 50 years. Treatment advancements with immunotherapies and targeted therapies over the past decade have contributed significantly, particularly with use of combination therapies.^36^, ^37^, ^38^ Improved surveillance efforts have also been suggested as influential in reducing mortality rates of melanoma through the increased detection of thin melanomas.^39^

Our results show that factors associated with disease-specific mortality include increasing Breslow thickness, higher age at diagnosis, left anatomic site, and increasing AJCC V8 pathologic stage and are consistent with previous reports in the literature.^40^^,^^41^

Nodular melanoma was the only histogenic subtype found to have a statistically significant increased risk of death due to melanoma. This is consistent with a 2015 study in Europe that demonstrated the lowest survivorship associated with nodular histogenic subtype (highest with lentigo maligna subtype).^41^ Very thick melanomas (>4.00 mm) have been shown to be disproportionately represented by the nodular subtype^42^ which supports the idea that different histogenic subtypes have variations in biologic behavior and expected natural histories. It is possible that Breslow thickness and nodular subtype are confounding variables with respect to melanoma-specific mortality, but our findings further suggest that nodular melanomas present a higher risk of mortality.

Our data also demonstrate a 1.98-fold increase in melanoma-specific mortality associated with left-sided melanomas. Previously discussed theories to explain the higher incidence of left-sided tumors can also be considered to explain the observed association with mortality (increased UV exposure from driving,^31^^,^^32^ asymmetric embryonic development of melanocytes); however, these postulations explain elevated incidence, rather than mortality.^33^^,^^35^ This relatively high HR warrants further exploration into left-sided melanoma tumorigenesis.

Disease-specific mortality associated with male sex was found to be elevated among a univariable calculation (HR 2.00, *P* = .001) but when calculated using a multivariable analysis, this risk was reduced and not statistically significant (HR 1.53, *P* = .107). This suggests that confounding variables may influence the observed increased mortality among male sex such as histogenic subtype, Breslow thickness, and anatomic location.

Anatomic site was not associated with a statistically significant difference in mortality, consistent with previous reports.^41^ Race was also found to have no significant association with melanoma-specific mortality. The authors suggest a potential limitation in the dataset’s statistical power with only 2.2% of melanomas identified in non-White patients.

### Limitations

Our study is limited by the focused nature of the REP which tracks residents of Olmsted County, Minnesota. While this population tends to have a similar socioeconomic status to the greater United States, the population is largely non-Hispanic Caucasian and not racially representative of other demographics. The retrospective nature of chart review is inherently limiting. This study did not assess for UV exposure history, family history, or genetic influences as factors for melanoma incidence and mortality. Melanoma incidence and mortality among patients <18 was not assessed.

## Conclusion

The incidence of primary cutaneous melanoma in Olmsted County, Minnesota has increased steadily over the last 50 years, with a continued overall increase of 1.21-fold over the past 10 years. The highest increase over the past decade is among females aged 40 and older, with a relative stabilization among males. This divergence in sex incidence may indicate other external factors are contributing beyond overdiagnosis. Death due to melanoma has declined steadily over the past 50 years, with patient characteristics of age at diagnosis, nodular histogenic subtype, Pathologic stage, left anatomic site, and Breslow thickness most strongly associated with mortality. Although this study observes a confined subset of the US population, the granular, high-quality nature of the data with reliable long-term follow-up is important in determining trends in incidence and melanoma.

## Conflicts of interest

None disclosed.
